# Supplementary material for: Perceptual judgments are resistant to the advisor’s perceived level of trustworthiness: A deep fake approach
Source: PLoS One. 2025 Apr 16;20(4):e0319039. doi: 10.1371/journal.pone.0319039 (PMC12002497; doi:10.1371/journal.pone.0319039)
Supplement: S2 Table — (DOCX) [file pone.0319039.s002.docx]

**S2 Table**

*Note.* Model summary for the analyses of the advice alignment rate. In the first column, you can find the different variables. In the second column, you can find the beta coefficients. In the third column, you can find the standard error. In the fourth column the z.values and in the last column the corresponding p-values. The variables are coded according to sum coding, with untrustworthy as the reference level (-1). The other level is coded as 1. The intercept represents the grand mean.

| **Model Summary For The Advice Alignment Rate (logit)** | | | | |
| --- | --- | --- | --- | --- |
| *variables* | *beta* | *se* | *z.value* | *p.value* |
| (Intercept) | 1.31 | 0.05 | 24.91 | < .0001 |
| trustworthiness1 | -0.03 | 0.02 | -1.20 | .230 |
