# Supplementary material for: Perceptual judgments are resistant to the advisor’s perceived level of trustworthiness: A deep fake approach
Source: PLoS One. 2025 Apr 16;20(4):e0319039. doi: 10.1371/journal.pone.0319039 (PMC12002497; doi:10.1371/journal.pone.0319039)
Supplement: S3 Table — (DOCX) [file pone.0319039.s003.docx]

**S3 Table**

*Note.* Descriptives estimated marginal means for the choice decision time. In the first column, you can find the variable advice alignment (i.e., aligned, not aligned). In the second column, you can find the different levels of trustworthiness (i.e., trustworthy, untrustworthy). In the third column, you can find the estimated marginal mean for the choice decision times in milliseconds. In the last column, you can find the standard error.

| **Descriptives Estimated Marginal Means Choice Decision Time** | | | |
| --- | --- | --- | --- |
| *advice alignment* | *trustworthiness* | *mean (ms)* | *se* |
| not aligned | trustworthy | 1587.31 | 32.74 |
| not aligned | untrustworthy | 1587.75 | 32.71 |
| aligned | trustworthy | 1327.44 | 23.69 |
| aligned | untrustworthy | 1328.10 | 22.82 |
