# Supplementary material for: Perceptual judgments are resistant to the advisor’s perceived level of trustworthiness: A deep fake approach
Source: PLoS One. 2025 Apr 16;20(4):e0319039. doi: 10.1371/journal.pone.0319039 (PMC12002497; doi:10.1371/journal.pone.0319039)
Supplement: S7 Table — (DOCX) [file pone.0319039.s007.docx]

**S7 Table**

*Note.* Table of the fixed and random effect structure for our exploratory analyses in Experiment 2 including difficulty. In the first block you can find the dependent variables, in the second column you can find the fixed effects, and in the last column you can find the random effect structure.

| **Models Experiment 2 Including Difficulty** | | |
| --- | --- | --- |
| *model* | *Fixed* | *Random* |
| Advice alignment rate | Trustworthiness*Difficulty | (Difficulty\|subject) |
| Choice decision time | Trustworthiness*Advice Alignment*Difficulty | (Advice Alignment+Trustworthiness+Difficulty\|subject) |
| Confidence ratings | Trustworthiness*Advice Alignment*Difficulty | (Difficulty\|subject) |
