# Supplementary material for: Perceptual judgments are resistant to the advisor’s perceived level of trustworthiness: A deep fake approach
Source: PLoS One. 2025 Apr 16;20(4):e0319039. doi: 10.1371/journal.pone.0319039 (PMC12002497; doi:10.1371/journal.pone.0319039)
Supplement: S8 Table — (DOCX) [file pone.0319039.s008.docx]

| **Descriptives Estimated Marginal Means Advice Alignment Rate Including Difficulty** | | | |
| --- | --- | --- | --- |
| *difficulty* | *trustworthiness* | *mean (%)* | *se* |
| easy | trustworthy | 98.83 | 0.28 |
| hard correct | trustworthy | 78.32 | 0.99 |
| hard incorrect | trustworthy | 39.41 | 1.94 |
| easy | untrustworthy | 98.64 | 0.32 |
| hard correct | untrustworthy | 79.26 | 0.96 |
| hard incorrect | untrustworthy | 38.23 | 1.92 |

**S8 Table**

*Note.* Descriptives for the estimated marginal means for the advice alignment rate. In the first column, you can find the difficulty levels (i.e., easy, hard correct, and hard incorrect). In the second column, you can find the trustworthiness levels (i.e., trustworthy, untrustworthy). In the third column, you can find the marginal estimated mean advice alignment rate (%), and in the last column the standard error.
