# Supplementary material for: Perceptual judgments are resistant to the advisor’s perceived level of trustworthiness: A deep fake approach
Source: PLoS One. 2025 Apr 16;20(4):e0319039. doi: 10.1371/journal.pone.0319039 (PMC12002497; doi:10.1371/journal.pone.0319039)
Supplement: S9 Table — (DOCX) [file pone.0319039.s009.docx]

| **Model Summary For Advice Alignment Including Difficulty (logit)** | | | | |
| --- | --- | --- | --- | --- |
| *variables* | *beta* | *se* | *z.value* | *p.value* |
| (Intercept) | 1.74 | 0.08 | 21.28 | < .0001 |
| trustworthiness1 | 0.02 | 0.03 | 0.75 | .453 |
| difficulty1 | 2.62 | 0.15 | 17.05 | < .0001 |
| difficulty2 | -0.43 | 0.07 | -6.15 | < .0001 |
| trustworthiness1:difficulty1 | 0.05 | 0.06 | 0.89 | .375 |
| trustworthiness1:difficulty2 | -0.05 | 0.03 | -1.51 | .132 |

**S9 Table**

*Note.* Model summary for the analyses of the advice alignment rate including difficulty. In the first column, you can find the different variables. In the second column, you can find the beta coefficients. In the third column, the standard error. In the fourth column, the z. values and in the last column the corresponding p-values. The variables are coded according to sum coding, with untrustworthy, and hard incorrect as the reference levels (-1). The other levels are coded as 1. The intercept represents the grand mean.
