# Supplementary material for: Perceptual judgments are resistant to the advisor’s perceived level of trustworthiness: A deep fake approach
Source: PLoS One. 2025 Apr 16;20(4):e0319039. doi: 10.1371/journal.pone.0319039 (PMC12002497; doi:10.1371/journal.pone.0319039)
Supplement: S10 Table — (DOCX) [file pone.0319039.s010.docx]

| **Descriptives Estimated Marginal Means Choice Decision Time Including Difficulty** | | | | |
| --- | --- | --- | --- | --- |
| *difficulty* | *trustworthiness* | *advice alignment* | *mean (ms)* | *se* |
| easy | trustworthy | not aligned | 1399.38 | 48.05 |
| easy | trustworthy | aligned | 1157.62 | 21.69 |
| easy | untrustworthy | not aligned | 1437.63 | 46.31 |
| easy | untrustworthy | aligned | 1162.28 | 21.74 |
| hard correct | trustworthy | not aligned | 1590.34 | 30.99 |
| hard correct | trustworthy | aligned | 1328.80 | 23.47 |
| hard correct | untrustworthy | not aligned | 1585.46 | 30.90 |
| hard correct | untrustworthy | aligned | 1329.43 | 23.24 |
| hard incorrect | trustworthy | not aligned | 1392.06 | 31.70 |
| hard incorrect | trustworthy | aligned | 1513.96 | 27.74 |
| hard incorrect | untrustworthy | not aligned | 1410.47 | 31.46 |
| hard incorrect | untrustworthy | aligned | 1498.70 | 27.61 |

**S10 Table**

*Note.* Descriptives for the choice decision time including difficulty. In the first column, you can find the different levels of difficulty (i.e., easy, hard correct, hard incorrect). In the second column, you can find the different levels of trustworthiness (i.e., trustworthy, untrustworthy). In the third Collum, you can find the different levels of advice alignment (i.e., aligned, not aligned). In the fourth column, you can find the estimated marginal means for the choice decision times. And in the last column, you can find the standard error.
