# Supplementary material for: Perceptual judgments are resistant to the advisor’s perceived level of trustworthiness: A deep fake approach
Source: PLoS One. 2025 Apr 16;20(4):e0319039. doi: 10.1371/journal.pone.0319039 (PMC12002497; doi:10.1371/journal.pone.0319039)
Supplement: S12 Table — (DOCX) [file pone.0319039.s012.docx]

| **Descriptives Estimated Marginal Means Confidence Ratings Including Difficulty** | | | | |
| --- | --- | --- | --- | --- |
| *difficulty* | *advice alignment* | *trustworthiness* | *mean* | *se* |
| easy | not aligned | trustworthy | 59.84 | 1.95 |
| easy | not aligned | untrustworthy | 65.25 | 1.85 |
| easy | aligned | trustworthy | 79.52 | 0.88 |
| easy | aligned | untrustworthy | 79.73 | 0.88 |
| hard correct | not aligned | trustworthy | 56.17 | 0.92 |
| hard correct | not aligned | untrustworthy | 56.89 | 0.92 |
| hard correct | aligned | trustworthy | 70.22 | 0.84 |
| hard correct | aligned | untrustworthy | 69.83 | 0.84 |
| hard incorrect | not aligned | trustworthy | 66.89 | 0.99 |
| hard incorrect | not aligned | untrustworthy | 66.77 | 0.98 |
| hard incorrect | aligned | trustworthy | 59.76 | 1.05 |
| hard incorrect | aligned | untrustworthy | 60.27 | 1.06 |

**S12 Table**

*Note.* Descriptives for the confidence ratings including difficulty. In the first column, you can find the different levels of difficulty (i.e., easy, hard correct, hard incorrect). In the second column, you can find the different levels of advice alignment (i.e., aligned, not aligned). In the third column, you can find the different levels of trustworthiness (i.e., trustworthy, untrustworthy). In the fourth column, you can find the estimated marginal means for the confidence ratings. And in the last column, you can find the standard error.
