# Supplementary material for: Perceptual judgments are resistant to the advisor’s perceived level of trustworthiness: A deep fake approach
Source: PLoS One. 2025 Apr 16;20(4):e0319039. doi: 10.1371/journal.pone.0319039 (PMC12002497; doi:10.1371/journal.pone.0319039)
Supplement: S13 Table — (DOCX) [file pone.0319039.s013.docx]

| **Model Summary For Confidence Ratings Including Difficulty** | | | | | |
| --- | --- | --- | --- | --- | --- |
| *variables* | *beta* | *se* | *df* | *t.value* | *p.value* |
| (Intercept) | 65.93 | 0.79 | 225.36 | 83.14 | < .0001 |
| trustworthiness1 | -0.53 | 0.23 | 17523.67 | -2.28 | .023 |
| advice alignment1 | -3.96 | 0.24 | 17651.25 | -16.34 | < .0001 |
| difficulty1 | 5.16 | 0.61 | 560.76 | 8.51 | < .0001 |
| difficulty2 | -2.65 | 0.31 | 615.35 | -8.58 | < .0001 |
| trustworthiness1:advice alignment1 | -0.47 | 0.23 | 17613.96 | -2.04 | .041 |
| trustworthiness1:difficulty1 | -0.87 | 0.42 | 17527.71 | -2.09 | .037 |
| trustworthiness1:difficulty2 | 0.44 | 0.25 | 17501.55 | 1.75 | .080 |
| advice alignment1:difficulty1 | -4.58 | 0.44 | 17323.94 | -10.47 | < .0001 |
| Advie alignment1:difficulty2 | -2.79 | 0.26 | 17501.66 | -10.58 | < .0001 |
| trustworthiness1:advice alignment1:difficulty1 | -0.83 | 0.42 | 17565.79 | -1.97 | .049 |
| trustworthiness1:advice alignment1:difficulty2 | 0.20 | 0.25 | 17601.80 | 0.78 | .436 |

**S13 Table**

*Note.* Model summary for the analyses of confidence ratings including difficulty. In the first column, you can find the different variables. In the second column, you can find the beta coefficients. In the third column, you can find the standard errors, and in the fourth column the degrees of freedom. In the fifth column, you can find the t-value, and in the last column the corresponding p-value. The variables are coded according to sum coding, with untrustworthy, aligned, and hard incorrect as the reference level (-1). The other levels are coded as 1. The intercept represents the grand mean.
