# Supplementary material for: Perceptual judgments are resistant to the advisor’s perceived level of trustworthiness: A deep fake approach
Source: PLoS One. 2025 Apr 16;20(4):e0319039. doi: 10.1371/journal.pone.0319039 (PMC12002497; doi:10.1371/journal.pone.0319039)
Supplement: Attachment — Submitted filename: Response to Reviewers.docx [file pone.0319039.s015.docx]

Reviewer #1: This study investigates how trustworthiness of social information affects perceptual judgements in human decision-making. They operationalize trustworthiness through deep fake generated faces/ They show that trustworthiness does not affect the judgment.

The study asks an interesting question, but I am unsure how valid trustworthiness here is. As the authors say in the Discussion that trustworthiness in the study is completely unassociated with the task at hand. I would say that it is unassociated with anything really, except for facial features. This connects to my biggest problem with the study – why are facial features used at all to operationalize trustworthiness?? What is the logic behind it? I suspect racist and sexist biases influence which facial features are deemed to be trustworthy or not.

Thank you for your insights. The facial stimuli used in this study were derived from a validated model designed to create faces that vary in perceived trustworthiness in a statistical and holistic manner. To the best of our knowledge, these social perceptions are not influenced by racist or sexist biases. Instead, research suggests that humans tend to overgeneralize subtle emotional expressions (Zebrowitz & Montepare, 2008), particularly in uncertain contexts (Oosterhof & Todorov, 2008), which are evolutionarily linked to approach and avoidance responses (Todorov, 2008; Todorov et al., 2015). Faces with positive valence are typically perceived as trustworthy, whereas those with negative valence are perceived as untrustworthy. This concept has been elaborated in the introduction (lines 116–129).

I like the Discussion – it addresses the limitations of the study quite well. A simple extension of this experiment would be to cultivate trustworthiness of advisers through either clearly showing initially whether the advice given is correct and incorrect by a particular person and then testing how people incorporate the advice based on trustworthiness. Or if the focus is on perceptual trustworthiness, then avtars that speak or gesture more confidently could be used to imply trustworthiness.

Thank you for this suggestion. Previous research has shown that manipulating the validity of advisors significantly affects advice following rates in both value-based (Van der Biest et al., 2020) and perceptual decision-making (Qi et al., 2018). While these studies demonstrate that knowledge-based trustworthiness manipulations influence decision-making, they cannot rule out the possibility that these effects are non-social, reflecting cue-following behavior rather than trustworthiness per se. When advice is consistently accurate, the optimal heuristic is simply to follow it and disregard inaccurate advice, regardless of social impressions. In the present study, we used perceptual manipulations to investigate how first impressions based on trustworthiness shape advice-following behavior without manipulating the validity of the received advice.

We agree that future research could explore the integration of multiple sources of perceptual trustworthiness cues, such as eye gaze duration, vocal tone, and gestures associated with confidence.

Can the authors show the different faces used for trustworthiness? I think there are 4?

Yes, these can be found on OSF (see line 197 and [OSF](https://osf.io/bu7dj/?view_only=5f8db5a766484b0f874947563deaf054)).

Minor point: Discussion lines 467-469 need to be rewritten for better sentence structure.

We have rewritten this section.

Reviewer #2: Research question and methods:

In this manuscript, Van der Biest et al. ask whether the trustworthiness of an advice giver impacts the advice taking rate of advice receiver on perceptual judgments. To that end, they conduct a random dot motion discrimination experiment where individuals have to indicate whether a patch of randomly moving dots move to right or left direction while they alter the percentage of dots that move coherently (coherence level). Crucially, this coherence level is adjusted to create two different levels of task difficulty as the authors hypothesize that the level of uncertainty in the environment interacts with trustworthiness and affects the advice taking behavior.

Although the question is very interesting and the study has merits, there are critical issues in the methodology and the statistical analysis that authors need to address in order for the results of the study to be clear. I will describe these issues in the following.

Methods:
- The authors measure the advice taking rate as the frequency of the trials that the participant has adhered to the decision of another person. This has a confound with participant’s own decision because the authors cannot tease apart the decision of the participant, in the absence of any advice, from the decision made by following the advice. In other words, it is not clear whether a participant’s decision was made by their own judgment or was influenced by the advice giver. Authors should connect additional data with which there are trials or blocks where the participants do not receive any advice, and they have to make decisions based on their own judgment. This creates a baseline to compare the own’s decision with the decision to adhering to the advice.

Thank you for this insight. The paradigm we employed is conceptually similar to that of Qi et al. (2018; see lines 110-112, 301-303, 477–485), who investigated how task reputation influences perceptual decision-making. Their findings demonstrated that task reputation affects whose advice participants follow or ignore. The goal of our experiment was to show differences in advice following rates when receiving advice from perceived trustworthy versus untrustworthy advisors, independent of participants' actual accuracy or overall advice following rates.

We agree that it is important to confirm that participants utilize the provided advice, but we argue that an additional experiment is unnecessary. In our study, we included both hard trials with correct advice and hard trials with incorrect advice. Given that both trials have the same coherency levels (i.e., 5-10%), the advice alignment rate should be the same (see our comments on your suggestion to change the term advice following rate). However, if the advice alignment rate, is different this would suggest that they are influenced by the advice that they receive. In other words, if participants are not influenced by the advice received, their performance on hard trials should be equally good or bad, regardless of whether the advice was correct or incorrect.

Therefore, we statistically investigate whether the advice alignment rate was different in these two conditions (i.e., hard correct advice vs hard incorrect advice). To test this, we used a linear mixed model and compared the advice alignment rate in both types of trials (i.e., hard correct, and hard incorrect). Please note that only for the hard trials with incorrect advice, we inverted the advice alignment rate. As aligning with the advice is in this case the wrong answer. Thus, in the hard trials with incorrect advice, the correct answer was the opposite of the received advice.

Our results demonstrated that the advice alignment rate was significantly different in both types of trials, which suggests that participants did use the advice they received (*χ²2* = 331.17, p < .0001). The probability of aligning with the advisor was 2.36 times, CI [1.87,2.97] higher during hard trials with correct advice (79%, 95% CI [77, 81]) compared to hard trials with incorrect advice (61%, 95% CI [58, 64]).

This significant difference suggests that participants are influenced by the advice that they receive. However, this is not influenced by the perceived trustworthiness of the advisor.

- I wonder why the design of the experiment is in a way that all trials with easy coherence involve correct advice instead of having both correct and wrong advice in all coherence levels.

If advisors made mistakes on easy trials, this would likely have affected their perceived trustworthiness. Specifically, in easy trials where the direction of dot movement is clear and there is little to no uncertainty, providing incorrect advice would influence both the advice-following rate (Van der Biest et al., 2024) and perceived trustworthiness (Chang et al., 2010). We have included this motivation in the manuscript (lines 300 - 304).

- I am not sure about the rationale behind picking up the coherent levels in the RDM task, but 55%-75% of motion coherence is usually considered very high and make the task very easy. Usually, values between 0 and 24% are selected for the motion coherence level because participants usually reach a very high performance level around 24% coherence level.

We thank the reviewer for highlighting this point. In our study, we adopted the terminology of the ROK-plugin (Strittmatter et al., 2023), where a coherency of 50% indicates that the stimuli are ambiguous (e.g., 50% of the dots move to the right, and 50% to the left). Consequently, a coherency of 55% means that 5% of the dots are coherently moving in one direction (left or right). Our coherency levels align closely with the literature and the reviewer’s suggestions. Specifically, for hard trials, the coherency ranged from 5–10%, while for easy trials, it was set at 25%.

We have revised the manuscript to incorporate the terminology recommended by the reviewer (e.g. , 142, 299)

I think the performance is essential to check and report in the two conditions because it might be the case that the performances are very high in this task (due to picking up the 55%-75% coherence) and therefore, the desired interaction that authors expected between trustworthiness and uncertainty has not been observed (because people are usually more susceptible to the opinion of others when there is uncertainty in the environment).

Building on our response to the previous comment, we argue that task difficulty was not the reason for the absence of an interaction. For hard trials, the coherency ranged between 5–10%, and our data showed that on hard trials with incorrect advice, participant accuracy (influenced by the advice) was only 61%. This indicates that the task difficulty was consistent with prior research and sufficiently challenging to investigate the hypothesized interaction.

Technical issues:
- The axis label needs to be changed whenever it says “advice following rate” because due to the same issue mentioned above, the authors cannot tease apart choice following advice from one’s own decision.

Thank you for this suggestion. We have changed the term advice following rate to advice alignment rate. We have changed this throughout the manuscript, appendix, and supplementary materials.

- What is the accuracy rate of participants? I think it is essential to have a violin plot of the accuracies with individual dots representing the accuracy of each individual and to make sure that the participants do not reach a ceiling level of accuracy.

We estimated participant accuracy based on performance in the hard trials with incorrect advice, where accuracy was approximately 61%, which is most likely deflated by the received advice. For the hard trials with correct and easy trials, the estimated accuracy is most likely inflated by the received advice.

To visualize the data, we created a violin plot displaying individual data points for advice alignment rates across easy trials, hard trials with correct advice, and hard trials with incorrect advice.

**Fig 1. Violin Plot of the Advice Alignment Rate (%) by difficulty**

*Note.* Graphical depiction of the individual advice alignment rates for each participant. On the x-axis you can find the advice alignment rate, and on the y-axis the different difficulty levels (i.e., easy, hard correct, and hard incorrect). Each dot represents an individual participant. Especially for the hard correct trials, we can see that the data is following a normal distribution.

Upon closer inspection of the data, only 15 out of 199 participants agreed with the advice on more than 70% of trials, indicating no ceiling effect for participant alignment rate (or accuracy). Additionally, we clarified in the manuscript that the coherency in the hard trials was 5–10% (see response to the previous comment), which made the task sufficiently challenging. As a result, a ceiling effect is unlikely.

- In supplementary materials, the analyses have been separated into easy, hard, and incorrect advice. While it is clear from the text that the incorrect advice happen during the hard trials only, it is necessary to put the labels correctly (i.e., easy correct, hard correct, hard incorrect).

Thank you for this suggestion. We have changed the labels of the figures in the appendix, as well as in the tables and the text.

- Is the RT in the main text the average of RTs across the three conditions in the supplementary materials? If yes, why do we see an opposite trend in most of the trials (easy and hard) in the supplementary materials?

All reported results in the manuscript are the marginal estimated means according to the respective model for the hard trials with correct advice. In the appendix, we repeated the analyses with difficulty as a factor. However, we have realised that the legend of the Fig 1 in the appendix is incorrect. We have changed the colour scheme so that it is in accordance with our manuscript. Thank you for noticing this.

- While it is interesting to use deep fake technology to come up with more realistic social stimuli, the faces of the advice givers in the experiment are not natural, making the participants less susceptible to be influenced by them. Thus, I am not sure about the real use of the deep fake here.

We used computer-generated faces to enable statistical manipulation of perceived trustworthiness. By applying deep fake models to these static faces and demonstrating that their animated versions elicit differences in perceived trustworthiness, we provide a robust foundation for future first impression research. That said, we agree that future studies could move beyond computer-generated faces to incorporate real faces or investigate the impact of social characteristics in real-life settings.

- The flow of the manuscript in the introduction seems like a post-hoc analysis at some parts. For example, the authors hypothesize that they would expect to see and interaction between trustworthiness and advice following for decision time while they do not mention anything about why they expect this interaction in the literature review.

We have thoroughly revised the manuscript to improve readability and strengthen the rationale for our hypotheses, grounding it firmly in the existing literature (see lines 68–165).

Statistical analyses:

- The authors have used linear mixed models to predict advice taking rate, but they have not done any model comparisons. For example, why the confidence rate is not a variable in the linear mixed model (as the authors also mention in the introduction that it is a variable that influences the advice taking)? The same model comparison issue also applies to the confidence and response time modeling.

Our paradigm and analyses were designed to address our predefined research questions. Since confidence ratings were measured after decisions were made, including them as a predictor of advice alignment —which are measured beforehand—would not provide meaningful insights. The same reasoning applies to choice decision times. As a result, we had no prior hypothesis regarding the confidence ratings for the advice alignment rate or the choice decision time.

To clarify, we determined the random effect structure using a backward selection procedure, which inherently involves comparing different models.

- It would also be interesting to look at the effect of current trials’ confidence on the next trial advice taking rate as the advice taking might show itself on the confidence history of the participants.

We thank the reviewer for this suggestion. While the proposed analyses are beyond the original scope of this paper, which focuses on the influence of perceived trustworthiness on decision-making, we agree that these analyses could provide valuable insights into how confidence affects advice-following behavior. Consequently, we conducted the suggested analyses. Numerically, the results indicated that when participants were confident on the current trial, they were less likely to follow the advice on the subsequent trial (Fig. 1). However, this effect was not significant, *χ²(*1) = 2.11, *p* = .147. Additionally, caution is warranted in interpreting these results, as the randomization of trial types means that advice alignment behavior is influenced not only by confidence on the previous trial but also by the difficulty of the current trial (and there were not enough observations to test this interaction).

**Fig 1. Advice alignment rate current trial depending on the confidence on the previous trial**

*Note.* Graphical depiction of the advice alignment rate on the current trial, depending on the confidence ratings of the previous trial. On the y-axis you can find the advice alignment rate of the current trial. On the x-axis you can find the reported confidence ratings on the previous trial. We have scaled the data with the scale function in R.

- The authors mention that they only focused on the hard trials with correct advice for their analysis, but I wonder why they did not consider hard trials with wrong advice too as they also could be the high uncertain conditions (even maybe more than correct trials, because correct trials are already in alignment with participant’s decision).

We did not include hard trials with incorrect advice in our primary analyses due to the limited number of such trials—only 16 in total (8 trustworthy, 8 untrustworthy). Given this small sample size, drawing strong conclusions from these analyses would not be appropriate. However, as detailed in the appendix, we reanalyzed our data with difficulty included as a fixed effect.

Writing:
- In general, the manuscript has room for improvement in the writing. I recommend the authors to revise the manuscript for clarity.

We reviewed the manuscript thoroughly and sought editorial feedback from a colleague, which led to significant improvements in the manuscript.

- This is minor but I believe that the format that the current introduction has been written could be changed to convey less redundant information, and to make it clearer what each paragraph is talking about. As an example (but not just limited to this one), the last paragraph begins with redundant information with the previous paragraph which could be moved there.

We thank the reviewer for the opportunity to revise our manuscript. Significant portions of the manuscript have been rewritten, and all changes are highlighted in green.
