# Supplementary material for: Perceptual judgments are resistant to the advisor’s perceived level of trustworthiness: A deep fake approach
Source: PLoS One. 2025 Apr 16;20(4):e0319039. doi: 10.1371/journal.pone.0319039 (PMC12002497; doi:10.1371/journal.pone.0319039)
Supplement: Attachment — Submitted filename: Response to Editor.docx [file pone.0319039.s016.docx]

**Specifically, we believe that the results of some of your analyses highlighted in response to Reviewer #2 are critical for understanding and appreciating the main findings and should be incorporated into the main manuscript. T**

**These include: 1. Figure 1 from the Appendix and the accompanying text should be included in the main manuscript as Figure 4 (though the tables can remain in the Appendix).**

**We have incorporated the Appendix into the manuscript, including the results section and figure (Lines 422-532). However, please note that our focus has been on the main and interaction effects involving difficulty, as these are the primary analyses of interest. Consequently, we have not included the main effects of trustworthiness or advice alignment rate, nor their interaction in the manuscript nor in the figure (only figures A-C are included). Additionally, the tables from the Appendix have been moved to the supplementary materials, and as a result, the Appendix can now be removed from the manuscript.**

"**2. The argument against a ceiling effect (from your rebuttal, starting with "Upon closer inspection of the data...") should also be included in the main text. Related to the this, the accuracy rate for different conditions should be reported in the main text.**

**We incorporated this section directly following our difficulty analyses (Lines 512 - 526). Please note that the results in the manuscript (***χ²_2_* = 330.1, *p* < .0001**) slightly deviate from the results reported in our previous response letter (χ²_2_ = 331.17, p < .0001). There were no differences in the marginal estimated means, nor did this change the interpretation of the results. We discuss this control analyses in the general discussion (Lines 548 - 549).**

**3 . The results on alignment rate as a function of confidence rating from previous trials are interesting and should be reported in the main text, even if the results are not statistically significant.**

**Thank you for this suggestion. We have reanalyzed the data, including all trials as well as only hard trials. The z, and p-value (*χ²_1_* = 2.40, *p* = .122) slightly deviates from our previous response letter (*χ²_1_* = 2.11, *p* = .147). There was no difference in the marginal estimated means. Furthermore, this does not change the interpretation of the results. We have incorporated these analyses into the manuscript (Lines 527 -532).**
